# Primary Open-Angle Glaucoma Is Associated with Short-Term Memory Decline and Dementia in Individuals of African Ancestry

**DOI:** 10.3390/jcm13144140

**Published:** 2024-07-16

**Authors:** Tzuriel Sapir, Patrick Augello, Roy Lee, Makayla McCoskey, Rebecca Salowe, Victoria Addis, Prithvi Sankar, Gui-Shuang Ying, Joan M. O’Brien

**Affiliations:** 1 Department of Ophthalmology, Scheie Eye Institute, University of Pennsylvania, Philadelphia, PA 19104, USA; 2 TOC Eye and Face, Austin, TX 78705, USA

**Keywords:** cognitive impairment, glaucoma, vision, T-MoCA, Montreal Cognitive Assessment, dementia, African American

## Abstract

**Background**: Over the last decade, studies have suggested that primary open-angle glaucoma (POAG) may be associated with cognitive impairment and dementia, as both pathologies are age-related neurodegenerative processes. It remains unclear to what extent neurodegeneration in POAG extends to other neurological functions beyond vision, such as cognition. This follow-up study examined the potential association between POAG and cognitive decline in an African ancestry population. **Methods**: The Telephone-Montreal Cognitive Assessment (T-MoCA) was administered to POAG cases and controls previously enrolled in the Primary Open-Angle African American Glaucoma Genetics (POAAGG) study. Cases were assessed for retinal nerve fiber layer (RNFL) thickness and for the presence of dementia via chart review. Comparisons between POAG cases and controls were performed using two-sample *t*-tests for the T-MoCA total score and five subsection scores, and using chi-squared tests for incidence of dementia. Current scores were compared to scores from this same cohort from 7 years prior. **Results**: The T-MoCA was administered to 13 cases and 20 controls. The mean ± standard deviation (SD) T-MoCA total score was 15.5 ± 4.0 in cases and 16.7 ± 3.5 in controls (*p* = 0.36). However, there was a borderline significant difference in the delayed recall sub-score (2.3 ± 1.6 for cases vs. 3.4 ± 1.5 for controls, *p* = 0.052) and a significant difference in its sub-domain, the memory index score (MIS, 9.1 ± 4.3 for cases vs. 12.1 ± 3.0 for controls, *p* = 0.02). There were no significant differences between cases and controls for the remaining subsections. During 7 years of follow-up, a higher incidence of dementia was noted in POAG cases (7.1% for cases vs. 0% for controls, *p* = 0.058). Over 7 years, there was no significant deterioration in the cognitive performance of cases versus controls, and no association was seen between RNFL thinning and cognitive impairment. **Conclusions**: In this small-sample follow-up study of African ancestry individuals, POAG cases demonstrated worse short-term memory and higher incidence of dementia compared to controls. Future larger studies are needed to further investigate the presence and impact of neurodegeneration in POAG.

## 1. Introduction

Glaucoma is a disease characterized by retinal ganglion cell (RGC) damage, optic nerve degeneration, and subsequent progressive vision loss. It is the leading cause of irreversible vision loss worldwide [1]. Primary open-angle glaucoma (POAG) is the most common form of the disease, with an estimated 68.56 million people affected worldwide [2]. Risk factors for development of POAG include older age, family history of disease, elevated intraocular pressure (IOP), and African ancestry [3].

In the last decade, it has been suggested that POAG may be associated with cognitive impairment, as both pathologies are age-related neurodegenerative processes. Several common risk factors and associated pathophysiologic mechanisms are shared between dementia and POAG, including intracranial pressure changes and associated increased IOP, optic nerve and RGC degeneration, cerebrospinal fluid circulatory failure and sequestration, and the presence of both amyloid-β and tau proteins, known for their role in Alzheimer’s disease (AD) [4,5,6,7,8,9,10,11].

The association between POAG and cognitive impairment comes in lieu of a paradigm shift in POAG—from the view of POAG as a primary disease of the eye to a recognition of POAG as an ocular manifestation of overarching systemic dysregulation [5]. Factors beyond elevated IOP likely contribute to the pathogenesis of vision loss in POAG, as controlling IOP—while partially effective at slowing disease progression—fails to prevent continued visual deterioration in up to 37% of POAG patients [12,13,14,15,16,17]. Furthermore, there is evidence that POAG is associated with lesions in the central visual pathway, including the lateral geniculate body and the primary visual cortex, and that several POAG risk loci are shared with other neurodegenerative disorders [18,19,20]. This has sparked interest in uncovering other targets for treatment for POAG patients, such as neuroprotective agents [21].

It remains unclear to what extent neurodegeneration in POAG extends to other neurological functions beyond vision, such as cognition. Studies have found that the prevalence of POAG was increased in patients with AD, and that patients with POAG are more likely to develop dementia [22,23,24,25,26]. This relationship has also been shown to be specific for POAG as opposed to primary angle-closure glaucoma (PACG), and normal-tension glaucoma (NTG) as opposed to high-tension glaucoma (HTG) [27,28]. In contrast, other studies have found minimal association between POAG status and risk of dementia or cognitive decline, or have been limited by small sample size or by the confounding of glaucomatous vision loss impacting cognitive assessment scores [29,30,31,32]. The association between POAG and cognitive decline remains particularly unclear and understudied in individuals of African ancestry, who demonstrate the highest incidence and most severe progression of POAG [33,34,35,36]. The predictive value of cognitive screening for dementia or AD in POAG patients has similarly remained undefined, leaving the clinical utility of such testing unclear.

Here, we investigated the association between POAG and cognitive impairment in African ancestry individuals, with the goal of determining whether POAG cases experience greater cognitive decline compared to healthy controls. Within the Primary Open-Angle African American Glaucoma Genetics (POAAGG) study cohort, we compared case and control scores on the Telephone-Montreal Cognitive Assessment (T-MoCA), a validated cognitive screening tool designed for those with visual impairment. We also evaluated whether prior MoCA scores from these same subjects were predictive of severe cognitive decline or a dementia diagnosis over a period of seven years [31,37]. In a topic that lacks longitudinal analyses of a historically understudied African ancestry population, the aim of this study was to determine if MoCA scores can serve as a biomarker for further cognitive decline and risk of dementia among POAG patients.

## 2. Materials and Methods

### 2.1. Study Population

Subjects for this study were recruited from the larger POAAGG study. Eligibility criteria for the POAAGG study include self-identification as Black (either African American, African descent, or African Caribbean) and an age of 35 years or older. Exclusion criteria include a history of glaucoma, iritis, uveitis, iridocyclitis, age-related macular degeneration (AMD), and advanced proliferative diabetic retinopathy. Subjects were recruited during regularly scheduled visits to the Ophthalmology Department at the University of Pennsylvania, as well as several nearby ophthalmology clinics and outreach events. Fellowship-trained glaucoma specialists or ophthalmologists categorized subjects as case, control, or suspect based on detailed clinical criteria [37]. Briefly, cases were defined as having an open iridocorneal angle and characteristic optic nerve defects with corresponding visual field loss, and controls exhibited no confounding ocular conditions. An extensive description of the POAAGG study design, baseline demographics, complete eligibility criteria, and phenotyping methods is found elsewhere [37]. All subjects provided informed consent prior to involvement. The study adhered to the principles of the Declaration of Helsinki and was approved by the University of Pennsylvania Institutional Review Board (IRB).

### 2.2. Prior Cognitive Assessment

We previously assessed and reported the cognitive function of 137 POAAGG subjects between June 2016 and August 2017 using the Montreal Cognitive Assessment (MoCA), a test developed to screen for mild cognitive impairment (MCI) [31]. The MoCA has previously been validated and shown to be sensitive and specific for the detection of MCI, with higher sensitivity for MCI than the Mini Mental Status Exam (MMSE) [38]. MoCA administration takes less than 15 min and assesses patients in seven domains of cognitive function: visuospatial/executive function, naming, attention, language, abstraction, delayed recall (which is further classified by a memory index score, MIS), and orientation. MoCA total score and sub-scores of each domain range from 0 to 30 points, with a lower score indicating worse cognitive function. The details, results, and conclusions of this sampling have been previously described [31].

### 2.3. T-MoCA Assessment

In this study, we re-approached POAAGG cases and controls who had completed a prior MoCA assessment (described above). Between June and November 2023, we called 137 subjects and invited each individual to undergo the T-MoCA. The T-MoCA is adapted for administration by voice only, by removing the visuospatial/executive function and naming sections of the test, which require visual abilities, but keeping five sub-domains: attention, language, abstraction, delayed recall (including MIS), and orientation. T-MoCA total score and sub-scores for each domain range from 0 to 22 points, with a lower score indicating worse cognitive function. T-MoCA is validated against in-person administration of the full MoCA, and T-MoCA and MoCA scores are significantly correlated and have similar sensitivity for detecting cognitive impairment identified through an extensive in-person cognitive evaluation [39,40]. We chose to use the T-MoCA, rather than the full MoCA that was previously used, to increase our reach of subjects, who can complete the assessment from the comfort of their homes, as well as to reduce the confounding effect of visual impairment between the case and control groups. Of the 137 subjects approached several times, 33 (13 POAG cases and 20 controls) participated in this study. A total of 21 declined to participate, 16 were reported deceased, and 67 could not be reached (did not answer phone, did not have updated contact information, etc.). One investigator (TS), masked to diagnostic status (case or control) and other ophthalmic testing data, administered the T-MoCA to all 33 subjects.

### 2.4. Demographic and Phenotypic Information

Demographic information, including age, sex, medical history, and social history, were obtained during standardized interviews during enrollment and augmented using electronic medical records. Each subject also underwent a thorough ophthalmological assessment to allow collection of ocular phenotypic information, including retinal nerve fiber layer (RNFL) thickness measurements from Optical Coherence Tomography (OCT). No additional OCT testing was conducted at the time of T-MoCA administration. Dementia history was verbally obtained during T-MoCA administration.

### 2.5. Statistical Analysis

Comparison between POAG cases and controls in their baseline characteristics and T-MoCA scores were performed using two-sample *t*-test for means, and a chi-squared test or Fisher’s exact test for proportions. Associations between baseline factors with total T-MoCA score were evaluated using generalized linear models. Spearman correlation coefficients (R) were calculated to assess correlation between RNFL thickness and T-MoCA scores and correlation between change of RNFL thickness and change of T-MoCA scores. All statistical analyses were conducted using SAS version 9.4 (SAS Institute Inc., Cary, NC, USA), and two-sided *p* < 0.05 was considered statistically significant.

## 3. Results

### 3.1. Population Characteristics

The T-MoCA test was administered to 13 POAG cases and 20 healthy controls. The mean age ± SD was similar between cases (74.2 ± 10.4 years) and controls (73.9 ± 12.9 years) (*p* = 0.94). Cases and controls were similar in other demographic and clinical characteristics (Table 1).

### 3.2. Demographic Characteristics Are Not Associated with T-MoCA Score Changes

In the univariate analysis, the mean T-MoCA total score was 15.46 in cases and 16.70 in controls (mean difference = −1.24, 95% CI = −3.96, 1.48, *p* = 0.36). No demographic characteristics were significantly associated with T-MoCA score (Table 2).

### 3.3. POAG Status Is Associated with Short-Term Memory Decline

When comparing T-MoCA subsection domain scores between POAG cases and controls, there was a borderline significant difference between cases and controls in the delayed recall domain (mean difference = −1.09, 95% CI = −2.20, 0.01, *p* = 0.052) and a significant difference in its sub-domain, the memory index score (mean difference = −3.02, 95% CI = −5.60, −0.44, *p* = 0.02). There were no significant differences between cases and controls for the remaining subsection domain scores (Table 3).

### 3.4. POAG Status Is Associated with Dementia

On chart review, during the seven-year follow-up after the initial MoCA assessment at baseline, 5 (7.14%) of 70 POAG cases and 0 of 67 controls developed dementia (*p* = 0.058, Table 4).

### 3.5. POAG Status Is Not Associated with Increased Rate of Cognitive Decline over Time

We compared the cognitive score difference between cases and controls in 2016–2017 (assessed by MoCA) and in 2023 (assessed by T-MoCA) for each of the sections assessed in T-MoCA. We observed no difference in these subsection scores between 2016–2017 and 2023 between cases and controls (Table 5).

### 3.6. Cognitive Decline in POAG Is Not Associated with Disease Severity

No significant association was found between RNFL thickness and T-MoCA score. Additionally, no significant association was observed between the change in RNFL thickness from 2016–2017 to 2023 and the change in each subject’s subsection score compared the mean subsection score (Table 6).

## 4. Discussion

This study sought to define the association between POAG status and cognitive decline in the present and over time in an African ancestry cohort. We found no significant difference in total T-MoCA score between cases and controls. However, there was a significant difference between cases and controls in the delayed recall subsection and its subclassification, the MIS. This section asked subjects to remember a list of five unrelated words, and to recall the list at the conclusion of the T-MoCA. Subjects were graded on the number of words correctly recalled and the use of hints. This result may suggest that certain cognitive abilities, such as the short-term memory tested in this section, may become impaired in POAG. While, to our knowledge, no other studies have directly examined the link between POAG and short-term memory loss, several studies have found an association between POAG and other forms of cognitive decline [25,31,41]. Our novel results, therefore, suggest that T-MoCA may serve as a tool for assessing short-term memory loss as a sign of early cognitive decline in the POAG population.

We further found a borderline significant difference between cases and controls in the diagnosis of dementia over the seven-year span of this study. These results are of particular interest given the work of Julayanont et al., which suggests that the MIS may serve as a predictor of conversion from MCI to AD [42]. Indeed, if low MIS score serves as a positive predictor for dementia, and if POAG status is associated with both a lower MIS score and a higher incidence of dementia, these results point towards a novel idea: in a POAG population, T-MoCA or MIS testing may be used to assess the risk of dementia and the relative indications for seeking neuroprotective treatment. To our knowledge, we are the first to report this novel finding.

Our results are also of interest given the association of glaucoma with AMD, and of AMD with dementia. Both POAG and AMD are neurodegenerative diseases that occur in the elderly and have a strong genetic component. These diseases have also previously been associated with each other, and both have independently been linked to cognitive decline [27,43,44,45,46]. This might suggest that there is a background driver of neurodegeneration shared among POAG, AMD, and dementia [47]. More research is needed on this topic.

Over time, there was no increased score disparity between cases and controls in each of the subsections tested on T-MoCA. Similarly, no significant association was seen between worsening disease severity (as measured by RNFL thickness) and worsening cognitive decline over seven years. In the literature, the relationship between RNFL thickness and cognitive abilities is similarly unclear [31,48]. There are several possible explanations for our results amidst this uncertainty. It is possible that neurodegeneration in POAG may not extend past the visual tract or globally affect neural pathways that moderate cognition. Alternatively, T-MoCA may not be a sensitive enough test to detect subtle differences in cognitive function in POAG. It is also possible that additional factors may affect cognitive decline in POAG, and that better-powered studies may be needed to define this observation.

This study has several limitations. This study consisted of an entirely African ancestry cohort. While POAG is more common and severe among African ancestry individuals, this population has been severely under-represented in POAG research, contributing to premature vision loss and subsequent economic and health outcomes. Therefore, there is a critical need for studies of open-angle glaucoma in this population. However, these results may not be applicable to other populations or reflect an association between POAG and cognitive function that is applicable to all patients [31]. Additionally, this study was limited by its small sample size and the inability to follow up with a substantial portion of the original cohort. Similarly, visual field data were not used as markers of disease progression due to the lack of high-quality data for all subjects of this small cohort study.

## 5. Conclusions

In conclusion, findings from this study suggest that there may be an association between POAG and worsening short-term memory and the development of dementia. Future studies are needed to further elucidate the relationship and clinical utility of cognitive testing in POAG.

## Figures and Tables

**Table 1:** Baseline demographic and clinical characteristics of POAG cases and controls.

Demographic	POAG Case (N = 13)	Healthy Control (N = 20)	*p*-Value
Age, Mean (SD)	74.2 (10.35)	73.9 (12.94)	0.94
Gender, N (%)			
Female	8 (61.5%)	15 (75.0%)	0.41
Male	5 (38.5%)	5 (25.0%)	
Level of Education, N (%)			
Completed Graduate Degree	0 (0.0%)	2 (10.0%)	0.10
Completed 4-Year Degree	5 (38.5%)	4 (20.0%)	
Some College	4 (30.8%)	3 (15.0%)	
Completed High School	1 (7.7%)	9 (45.0%)	
Less Than High School	3 (23.1%)	2 (10.0%)	
Diabetes, N (%)			
No	7 (53.8%)	13 (65.0%)	0.52
Yes	6 (46.2%)	7 (35.0%)	
Hypertension, N (%)			
No	2 (15.4%)	5 (25.0%)	0.51
Yes	11 (84.6%)	15 (75.0%)	
Smoking Status, N (%)			
Current Smoker	1 (7.7%)	4 (20.0%)	0.22
Former Smoker	7 (53.8%)	5 (25.0%)	
Never Smoker	5 (38.5%)	11 (55.0%)	

**Table 2:** Univariate analysis for associations between POAG status, demographics, and total T-MoCA.

Demographic	Level	T-MoCA Total Score: Mean (SD)	*p*-Value
POAG status	Case	15.46 (4.03)	0.36
	Control	16.70 (3.54)	
Gender	Female	16.57 (3.74)	0.42
	Male	15.40 (3.78)	
Education	Completed Graduate Degree	18.50 (2.12)	0.17
	Completed 4-Year Degree	18.22 (2.22)	
	Some College	16.00 (4.83)	
	Completed High School	15.40 (3.95)	
	Less Than High School	13.60 (2.88)	
Diabetes	No	16.25 (4.15)	0.94
	Yes	16.15 (3.13)	
Hypertension	No	17.57 (2.37)	0.28
	Yes	15.85 (3.98)	
Smoking status	Current Smoker	17.20 (3.56)	0.24
	Former Smoker	14.75 (4.31)	
	Never Smoker	17.00 (3.16)	

**Table 3:** Comparison of T-MoCA subsection domain scores between POAG cases and controls.

Category (Max)	Group	Mean (SD)	Mean Difference (Case—Control) (95% CI)	*p*-Value
Attention (6)	Case	4.38 (1.61)	0.08 (−1.05, 1.22)	0.88
	Control	4.30 (1.53)		
Language (3)	Case	1.62 (0.87)	0.02 (−0.72, 0.75)	0.97
	Control	1.60 (1.10)		
Abstraction (2)	Case	1.08 (0.86)	−0.37 (−0.92, 0.18)	0.18
	Control	1.45 (0.69)		
Delayed Recall (5)	Case	2.31 (1.55)	−1.09 (−2.20, 0.01)	0.052
	Control	3.40 (1.50)		
Memory Index Score (15)	Case	9.08 (4.31)	−3.02 (−5.60, −0.44)	0.02
	Control	12.10 (2.97)		
Orientation (6)	Case	5.77 (0.44)	0.42 (−0.23, 1.07)	0.20
	Control	5.35 (1.09)		

**Table 4:** Analysis of relationship between disease status and dementia diagnosis.

Disease Status	Dementia			*p*-Value
	No	Yes	Total	
Case	65 (92.86%)	5 (7.14%)	70	0.058
Control	67 (100%)	0 (0.00%)	67	
Total	132	5	137	

**Table 5:** Comparison of difference between POAG cases and controls on subsection domain scores in 2016–2017 and 2023.

Category (Max)	Group	Mean Difference (Case—Control)	*p*-Value
Attention (6)	2016–2017	−0.89	0.07
	2023	0.08	
Language (3)	2016–2017	0.15	0.70
	2023	0.02	
Abstraction (2)	2016–2017	0.15	0.17
	2023	−0.37	
Delayed Recall (5)	2016–2017	−0.43	0.37
	2023	−1.09	
Memory Index Score (15)	2016–2017	−1.64	0.44
	2023	−3.02	
Orientation (6)	2016–2017	−0.10	0.13
	2023	0.42	

**Table 6:** RNFL correlation to T-MoCA scores in 2023 and over time.

T-MoCA Section	Correlation between RNFL Thickness and T-MoCA Score in 2023 Spearman Correlation Coefficient (*p*-Value)	Correlation between Change in RNFL Thickness and Change in T-MoCA Score Compared to the Mean over 2016 to 2023 Spearman Correlation Coefficient (*p*-Value)
Attention	−0.01 (0.96)	0.38 (0.22)
Language	0.30 (0.31)	0.34 (0.27)
Abstraction	−0.37 (0.21)	−0.43 (0.17)
Delayed Recall	−0.03 (0.91)	0.15 (0.64)
Memory Index Score	−0.07 (0.82)	0.42 (0.17)
Orientation	0.20 (0.52)	−0.12 (0.71)
Total	0.07 (0.81)	0.24 (0.45)

## Data Availability

The data that support the findings of this study are available from the corresponding author upon reasonable request.
